# Parametric Study of Geometry and Process Parameter Influences on Additively Manufactured Piezoresistive Sensors Under Cyclic Loading

**DOI:** 10.3390/polym17121625

**Published:** 2025-06-11

**Authors:** Marijn Goutier, Thomas Vietor

**Affiliations:** Institute for Engineering Design, Technische Universität Braunschweig, 38108 Brunswick, Germany

**Keywords:** additive manufacturing, 3D printing, material extrusion, fused deposition modeling, piezoresistive sensor, conductive polymer composite, linearity, hysteresis, drift, repeatability

## Abstract

The additive manufacturing of piezoresistive sensors offers several advantages, such as the elimination of assembly or installation steps, enabling integration into complex parts precisely where desired, and compatibility with soft robotics applications. Previous studies have demonstrated that several characteristics of additively manufactured sensors, such as their resistance and sensitivity, are significantly affected by the selected printing parameters. This work seeks to further the understanding of the relationships between process parameters, material, sensor design, and the resulting sensor characteristics. To this end, sensors made from two materials, with differing printing layer heights, infill angles, and thicknesses, are characterized under cyclic tensile loading. For these sensors, the nonlinearity, hysteresis, and drift are analyzed. The findings indicate that both nonlinearity and hysteresis are significantly affected by the material choice, as well as the selected parameters. Notably, parameters that affect the sensitivity of the sensor, e.g., the infill angle, can have significant indirect effects on the nonlinearity and hysteresis errors. Through correct parameter selection, nonlinearity errors can be reduced by up to 30.7% or 25.3%, depending on the material used. The hysteresis error can be reduced by up to 38.7% or 23.8%, depending on the material. The drift over multiple cycles is found to be strongly material dependent, but can also be affected by the process parameters, e.g., the infill angle. Understanding the interactions between material, design, process, and the resulting sensor characteristics provides valuable insights for the successful design and additive manufacturing of piezoresistive sensors.

## 1. Introduction

Additive manufacturing (AM), sometimes also referred to as 3D printing, is a term that refers to a family of manufacturing processes that produce parts through the selective addition of material, typically in a layer-by-layer fashion. This approach offers several advantages when compared to traditional manufacturing processes. These advantages include the ability to produce highly complex parts without incurring a significant cost increase, and this complexity applies both to part geometry and the ability to combine multiple materials within a single part [1]. In recent years, this ability to produce multi-material parts has been exploited to achieve various forms of functional integration. Examples of such integrated functionality include conductive polymer composite-based conductors [2,3], actuators [4], resistive heaters [5,6,7], magnetically activated shape-memory functions [8], and various types of sensors, such as piezoelectric [9,10,11,12], capacitive [13,14,15], and piezoresistive sensors [16,17,18,19,20,21,22].

For the piezoresistive sensor type, relationships have been shown to exist between certain piezoresistive characteristics and both the sensor’s geometry and the additive manufacturing process parameters selected for its production. It has been demonstrated that these factors can affect a piezoresistive sensor’s unloaded resistance [23,24], as well as its sensitivity, as expressed by the gauge factor (GF) [20] or piezoresistive coefficient [25]. It has also been shown that the geometric freedom available when additively manufacturing sensors can be used to achieve desired sensor characteristics, for example, by using resistive part-shortening mechanisms at the macro- [21] or the meso-scale [26,27], both in fully additively manufactured sensors and in non-conductive parts that are subsequently metallized [28]. However, there are multiple characteristics of piezoresistive sensors for which the potential relationships with sensor geometry and process parameters have not yet been thoroughly investigated.

One such characteristic is nonlinearity in the sensor response. This behavior has been found to exist both in additively manufactured sensors [29,30,31,32] and in conductive polymer composites (CPCs) independently of additive manufacturing [33,34]. Hernandez et al. [30] compared the nonlinearity in additively manufactured sensors using two novel material formulations, with either 7.5 wt.% or 10.0 wt.% carbon nano fiber filler in a polylactic acid (PLA) matrix. Their findings showed a lower nonlinearity error for the material with 10.0 wt.% filler, which the authors attribute to different inter-filler distances. Different sensor lengths did not have a significant effect on this behavior, and further geometric variations or differing AM process parameters were not considered. A study by Gooding et al. [29] compared the nonlinearity error for additively manufactured piezoresistive sensors with varied width, thickness, and number of loops in a meandering shape. Their findings showed that only the thickness and a three-way interaction of all inputs were statistically significant for the resulting nonlinearity. Schouten et al. presented an approach to improve the linearity of additively manufactured sensors by using two oppositely loaded sensors [35], but did not investigate the influence of various process parameters or design choices on the nonlinearity. Although the suggested approach is effectual, it imposes limitations on the sensor design, both regarding the space requirement and regarding the fact that positioning sensors in such a way that they face opposite loads may not always be practically feasible. Such limitations mean that, although compensation strategies exist, minimizing the nonlinearity error in a single sensor configuration remains a desirable solution. Despite this, potential relations between design, process parameters, and the resulting sensor nonlinearity remain largely unexplored.

A further sensor property of interest is hysteresis, i.e., differing responses during the loading and unloading of the sensor. This property that has been shown to exist in additively manufactured sensors [19,29,31,36,37,38,39,40,41,42,43,44,45,46] and more generally in conductive polymer composites [47,48,49]. Gooding et al. [29] analyzed hysteresis in additively manufactured sensors made from a graphene-filled PLA. The work focused on the impact of changing the number of loops and the length of sensors with a meandering shape, as well as the influence of the build orientation and thickness. The authors stated that an increase in the number of end loops led to a decrease in hysteresis, although the effect was shown to not be statistically significant. The thickness of the gauge was found to have no effect on hysteresis for gauges printed in a non-flat orientation, whereas for the flat-oriented gauges an increase in thickness was associated with an increase in hysteresis. The authors attributed the latter to a loss of layer adhesion under load, rather than an effect of the gauge thickness itself. Other works have observed hysteresis in additively manufactured piezoresistive sensors but have not investigated the relationships between this behavior, AM process parameters, and sensor geometry.

Lastly, additively manufactured sensors have also been shown to exhibit drift when repeatedly loaded [29,50]. There typically is a trend in the resistance and the gauge factor during the first 10 cycles, after which the behavior stabilizes [51,52]. Existing literature has presented models and experiments detailing resistance relaxation behavior [53] and drift correction approaches [54] in non-additively manufactured CPC sensors. For additively manufactured sensors, drift was found to be significantly correlated to the sensor thickness and its interaction with width and the number of end loops in a meandering layout [29]. The work of Georgopoulou et al. [50] tested the drift behavior of sensors made from three different custom-made styrene–ethylene–butylene–styrene triblock copolymer materials. Their findings showed a significant difference between polymers with different Shore hardness levels, with the 50A material exhibiting higher drift than both the 25A and the 65A variants. The authors attribute these differences to the different matrix material compositions used to achieve the desired hardness levels. Although the existence of drift in additively manufactured piezoresistive sensors has been observed, potential relationships between this behavior and the additive manufacturing process parameters remain largely unexplored. Due to the aforementioned tendency for stabilization, it might be possible to bypass this phenomenon in certain practical applications by excluding the first cycles. For example, a robotic gripper with integrated sensors could be opened and closed repeatedly before the intended application commences. However, in other applications, e.g., structural health monitoring [55], such preconditioning possibilities may not exist. In these cases, it is important to understand how the behavior changes during the first loading cycles, and how this behavior may be impacted by the additive manufacturing process parameters.

Although the existence of nonlinearity, hysteresis, and drift errors has been observed, there has been limited investigation of the influences that AM process parameters and part geometry may have on those properties. Thus, the study of the different impact factors and their effects remains incomplete. Understanding how sensors’ characteristics are affected by their geometry and production process is important to enable the selection of suitable parameters to create performant sensors. Specifically, practical sensor implementations are significantly simplified when the sensor can be produced in a manner that minimizes error contributions from nonlinearity, hysteresis and drift. The current work aims to develop a deeper understanding of the influences exerted by geometry and AM process parameters by analyzing the relationships between these factors and sensor nonlinearity, hysteresis, and signal drift under repeated tensile loading. The parameters taken into consideration for this purpose are the printing layer height, the angle of the infill pattern, and the thickness of the sensor. Furthermore, two different conductive polymer composite materials are used to enable a comparison of material dependency of the observed effects.

## 2. Materials and Methods

This chapter provides an overview of the employed materials, the sensor geometry, and the additive manufacturing equipment and parameters used. Following this, the electromechanical measurement setup is presented. Lastly, a detailed definition is provided of the various error types that are analyzed in this work.

### 2.1. Sensor Design and Additive Manufacturing

**Figure 1:**
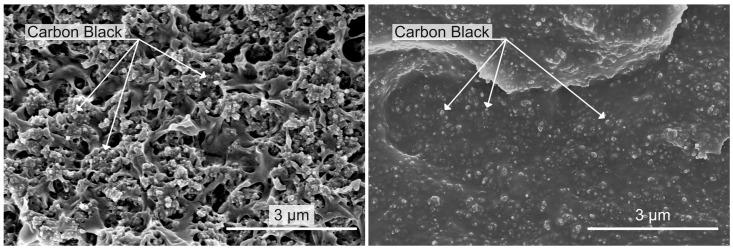
SEM micrographs of the cPLA (**left**) and the cTPU (**right**) filament, revealing only carbon black as the conductive filler.

The sensors investigated in this work are produced using two different commercially available conductive polymer composites. These are referred to as conductive polylactic acid (cPLA) and conductive thermoplastic polyurethane (cTPU). The former material is Proto-Pasta Conductive PLA, produced by Proto-Pasta (Vancouver, WA, USA), and has a manufacturer-claimed resistivity of 30 Ωcm on the X-/Y-plane and 115 Ωcm along the *Z*-axis [56]. The latter material is NinjaTek Eel, a thermoplastic polyurethane (TPU)-based material produced by Ninjatek (Lititz, PA, USA) that has a manufacturer-claimed resistivity of 1.5 × 10^3^ Ωcm [57]. Both of the CPCs use carbon black as the conductive filler, with a stated content of <21.43 wt.% for cPLA [58] and ≤18 wt.% for cTPU [59]. Previous literature has shown that some commercially available CPCs contain conductive fillers beyond those stated in their specifications [60]. The scanning electron microscopy (SEM) micrographs in Figure 1 reveal no conductive filler particles, other than the stated carbon black, in either of the CPCs used in this work.

**Figure 2:**
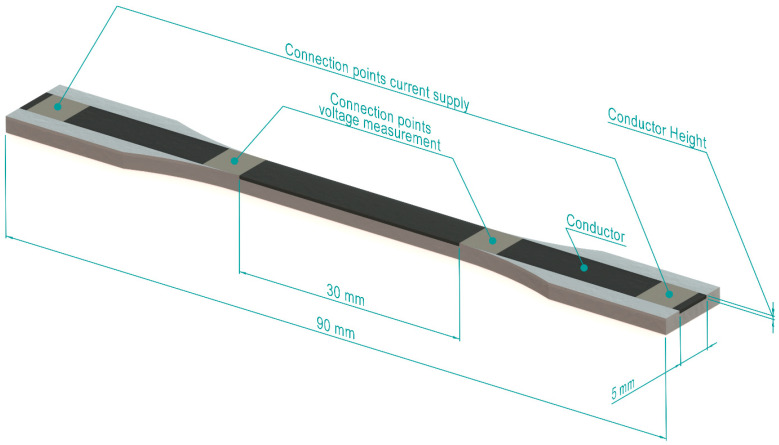
Rendering of a sensor, with the non-conductive TPU in white and the CPC sensor in black. The height of the conductive element is varied for different variants. Dimensions not listed are in accordance with ISO 20753, variant A22 [61]. The connection points indicate the locations from which a four-terminal resistance measurement was taken. Reproduced from Goutier et al. [20] and used under Creative Commons CC-BY license.

The sensors are integrated into a part with a geometry based on ISO 20753 [61], variant A22, as shown in Figure 2. This allows for the integration of different sensor thicknesses while maintaining constant overall dimensions. The surrounding geometry is created from Ninjatek NinjaFlex, a non-conductive thermoplastic polyurethane (TPU) [62].

The input factors for the experiment, aside from the two aforementioned conductive materials, are the printing layer height, the infill angle, and the conductor height, i.e., the thickness of the sensor. The printing layer height is set to either 0.1 mm or 0.2 mm. The infill is a rectilinear pattern with the angle varied between 0°, ±45°, or 90°, with 0° being the longitudinal direction of the sensor. The conductor height is varied between 0.2 mm, 0.4 mm, and 0.6 mm. No outlines or shells are used when printing the sensor, so the impact of the infill angle can be adequately assessed in isolation. For the non-conductive material, an infill angle of ±45° and layer height of 0.2 mm is used in all cases.

Potential influences of contact resistance are eliminated by using a four-terminal resistance measurement, the contact locations for which are shown in Figure 2. The distance between the two inner points is 30 mm. These inner two contact points are located in between the clamps that hold the sensor during tensile testing, eliminating unwanted piezoresistive effects due to clamping forces. To further ensure a reliable electrical contact, all contact locations are pre-treated with a colloidal silver ink (type 12640, Electron Microscopy Services, Hatfield, PA, USA). The outer locations are then contacted using pogo pins, while the inner locations are contacted by attaching copper wires using a conductive silver epoxy adhesive (8331-14G, MG Chemicals, Burlington, ON, Canada).

All specimen variants are produced in triplicate to ensure statistical reliability. The additive manufacturing is carried out on a ToolChanger and Motion System, equipped with Hermera direct extruders (E3D-Online Ltd., London, UK). The use of a setup with multiple independent toolheads follows the recommendations for the material extrusion-based additive manufacturing of sensors, as implemented in the work of Schouten et al. [63]. Hardened nozzles with a 0.4 mm diameter are used for all materials (Nozzle X, E3D-Online Ltd., London, UK). Material-specific settings, including the flow rate, extrusion speed, and extrusion temperature, are calibrated for each material and set at fixed values as per Table 1.

### 2.2. Measurement

Sensor resistance is measured in a four-terminal configuration using a Keithley 2750 Multimeter/switch system (Tektronix Inc., Beaverton, OR, USA). The tensile tests are performed on a Zwick Z0.5 axial materials testing machine (Zwick GmbH & Co., Ulm, Germany), with an accuracy class of 0.5 in accordance with ISO 7500-1 [64]. Simultaneous strain measurements are performed with a non-contact video extensometer (VideoXtens 1-120, Zwick GmbH & Co., Ulm, Germany). The test procedure is based on ISO 527-1 [65]. To eliminate potential thermoresistive influences, the measurements are performed in climate-controlled conditions.

Based on the allowable strain range while avoiding plastic deformation for sensors with these CPCs [20], the maximum strain is set to 0.75%. The strain rate is set to 2 mm/min, and the cycle count for each measurement is 10. These first cycles are of particular interest, because existing literature has shown a change in the resistance and the gauge factor to occur during the first 10 cycles, followed by stabilization [51,52].

### 2.3. Analyzed Properties

**Figure 3:**
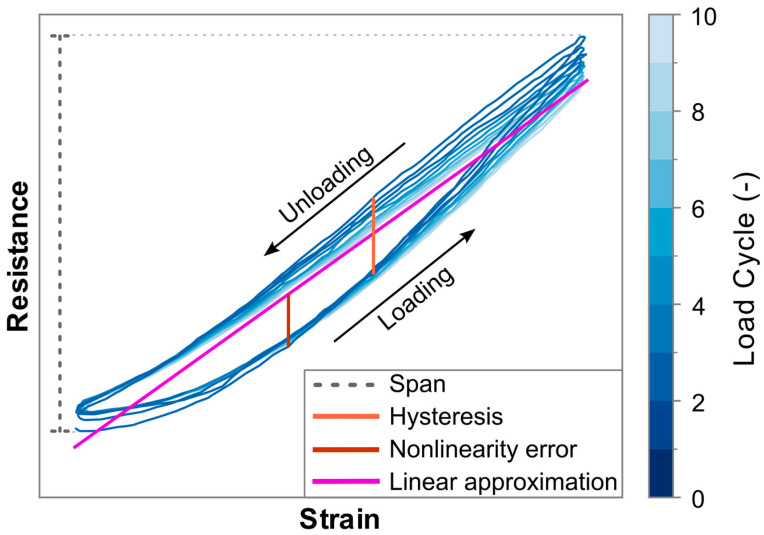
Example of a sensor resistance signal under cyclic load, color graded for the cycle count. The figure includes graphical representations of the span, the BFSL linear approximation of the signal, the nonlinearity error, and the hysteresis error.

This work analyzes the nonlinearity error, hysteresis error, and drift over multiple cycles. To allow for the contribution of the different error types to be compared among each other and also between different sensors and materials, each is expressed as a percentage of the span for each sensor. The span can be found by subtracting the minimum resistance value from the maximum resistance value during the measurements, as graphically represented in Figure 3. The error types are then defined as listed below.

**Figure 4:**
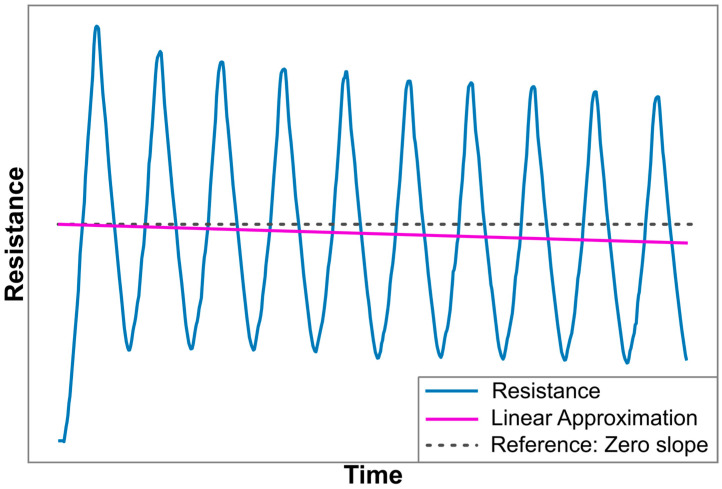
Graphical representation of a sensor resistance signal while a cyclic strain load is applied, and a linear approximation of that signal. The sensor in this example exhibits decreasing maximum resistances during the first cycles, which leads to a downwards trend in the linear approximation. The slope of this linear approximation can be used to evaluate the output drift, as demonstrated in [66]. The dashed line provides a visual reference of a zero-slope line.

- Nonlinearity error: The absolute maximum difference that occurs between a sensor’s resistance in response to strain, and a best fit straight line (BFSL) type of linear approximation of that signal. Both the BFSL and the nonlinearity error are depicted in Figure 3.
- Hysteresis error: The maximum difference between the upwards and downwards slopes of the resistance at any given strain level within a cycle, as shown in Figure 3.
- Drift over multiple load cycles: Analyzed using the approach as demonstrated in the work of Scholle et al. [66], which utilizes the slope of a linear approximation of the resistance over time signal, as illustrated in Figure 4. Because the load case for the experiments in the current work forms a symmetrical signal, with this approach a theoretical perfect piezoresistive material, without any drift, would exhibit a linear approximation with zero slope. A positive or a negative slope value indicates that the sensor response exhibits an increasing or decreasing trend in resistance under repeated loading, respectively. The values used in this work are the average increase or decrease per load cycle as a percentage of the span for that particular sensor. The approach of Scholle et al. is selected over similar ones used to characterize additively manufactured sensors by Georgopoulou et al. [50] or Gooding et al. [29], because the former considers only changes in the maximum values, disregarding potential drift in the minimum resistance values, while the latter analyzes the drift only for the first two cycles.

To systematically analyze how the three error types are affected by the infill angle, layer height, sensor thickness, and material, the program Minitab (version 22.2.1, Minitab LLC., State College, PA, USA) is used to create linear regression models for each error type. These models allow for the identification of statistically significant factors, and can also capture interaction effects. The values for nonlinearity error, hysteresis error, and signal drift are selected as the outputs for the linear regression models. The material, infill angle, layer height, and conductor height are used as the inputs. Of these, the material and the infill angle are taken as categorical variables, while the layer height and conductor height are set as continuous variables. Terms and interaction effects are included through the second order, and cross predictors are included. Data is standardized by subtracting the mean and dividing by the standard deviation. Insignificant terms are eliminated from the models through a process of backwards elimination.

## 3. Results and Discussion

### 3.1. Nonlinearity Error

**Figure 5:**
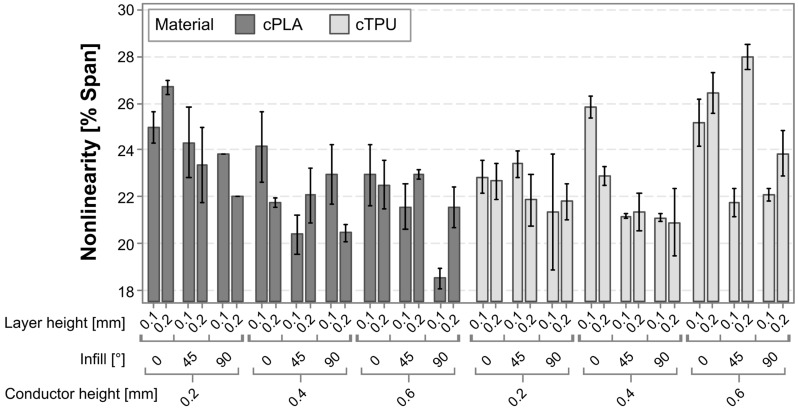
Bar chart of the linearity error for each of the tested sensor variants.

Figure 5 provides a graphical representation of the linearity error for each of the tested sensor variants. For both materials, a reduction in the error is typically visible as the infill angle is increased, although there are a few exceptions, e.g., 0.4 mm-thick cPLA at 0.1 mm layer height. For cPLA, there is also a reduction related to an increasing conductor height, which is not immediately recognizable for cTPU.

**Figure 6:**
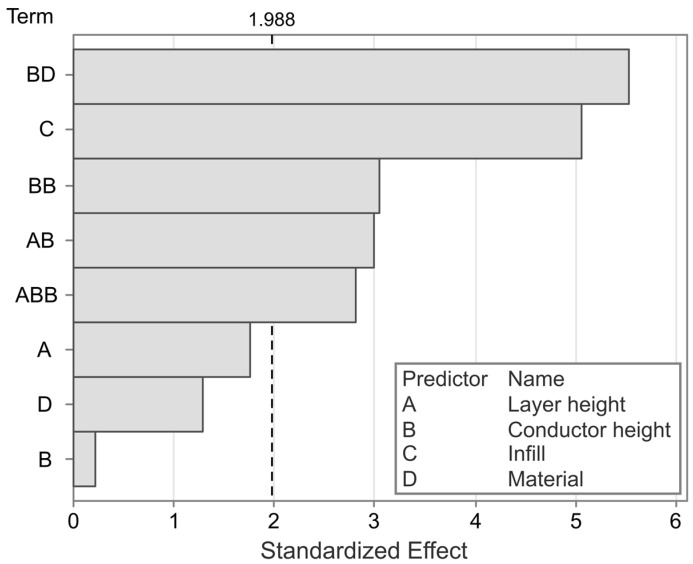
Pareto chart of the significant effects on the sensor nonlinearity. The dashed line indicates the a = 0.05 significance level.

To gain a better understanding of the contributing factors and interaction effects, and to assess their statistical significance, a linear regression model is employed. The model has an R^2^ value of 61.97%, indicating that most, but not all, influencing factors are captured in the experimental variables. The most significant factors in the model can be found in the Pareto chart, as shown in Figure 6.

**Figure 7:**
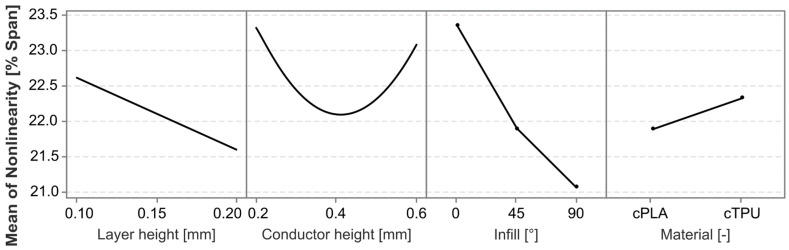
Main effects plot for the factors impacting the nonlinearity error.

**Figure 8:**
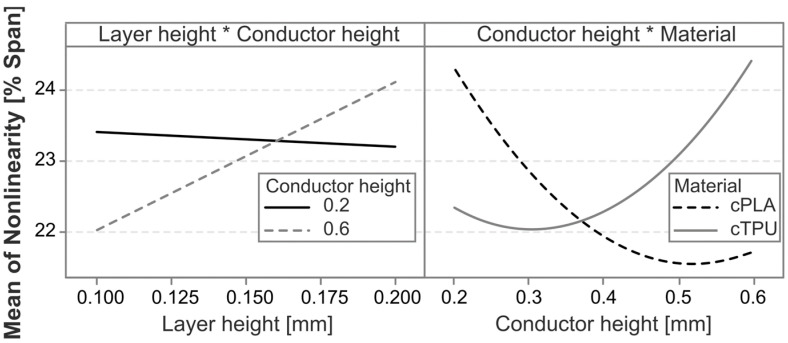
Interaction plots for the statistically significant interaction effects on the nonlinearity error. The * symbol indicates an interaction between the two factors.

The most significant factors affecting sensor nonlinearity are the interaction between material and conductor height, followed by the infill angle, which is in agreement with the observations made from Figure 5. The exceptions regarding the effect of the infill angle, which are observed in Figure 5, are not statistically significant effects or interaction effects. Other significant effects are the conductor height squared, and the interaction effects between the layer height and conductor height (squared). The individual factors of layer height, material, and conductor height are not themselves statistically significant, but are retained to ensure model hierarchy. To further analyze the different effects and their directionality, the main effects and interaction effects are plotted in Figure 7 and Figure 8, respectively.

The nonlinearity error differs between the two tested materials, with cPLA showing a slightly lower mean nonlinearity error than cTPU. The difference is particularly pronounced in the interaction between the material and the conductor height, with cPLA reaching its lowest error value at greater conductor heights than cTPU. As the two CPCs have different filler concentrations, this behavior could be related to the increased inter-filler distances in cTPU when compared to cPLA, similar to the findings of Hernandez et al. [30]. The resistivity in cTPU also increases more rapidly with the layer count than it does in cPLA, due to a pronounced layer-to-layer interface effect in additively manufactured cTPU [20]. This causes parts with a greater height, and therefore a greater number of layers, to show an increase in resistivity [24]. The layer-to-layer interfaces likely have a locally reduced percolation network density, which may lead to increased nonlinear behavior under strain.

**Figure 9:**
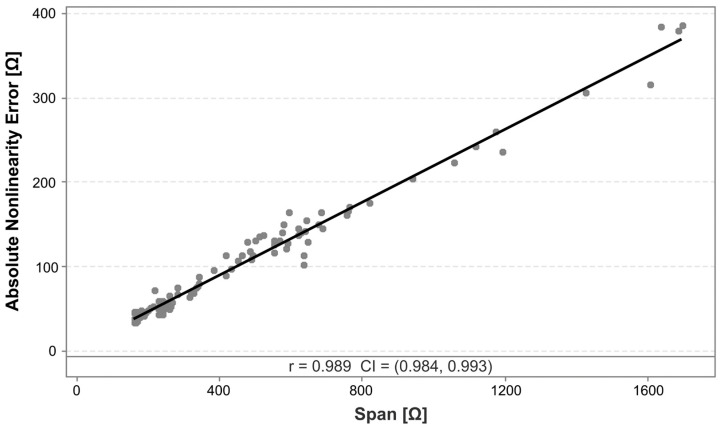
Scatterplot of the correlation between the absolute nonlinearity error and the span, including a linear regression line of the effect. The indicated values for r and CI represent the Pearson correlation coefficient and the 95% confidence interval, respectively.

The effect of the infill angle is significant, showing a strong reduction in the nonlinearity error for both materials as the infill angle is increased. Notably, this effect appears to be the inverse of the influence that the infill angle has on the sensor sensitivity, as expressed by the gauge factor [20]. It is therefore possible that an increase in the infill angle leads to an increased sensitivity, which results in a larger span for a given level of strain. Equation (1) [67] describes the relationship between the gauge factor and the span.

(1) $$GF=\frac{\Delta R/R_{0}}{\Delta l/l_{0}}=\frac{\Delta R/R_{0}}{\varepsilon }$$

where *GF* is the gauge factor, Δ*R* is the change in resistance under load, *R*_0_ is the initial unloaded resistance, Δ*l* is the change in length under load, *l*_0_ is the unloaded sensor length, and ε is the strain. It can be seen that the span, i.e., the maximum value for Δ*R*, is a function of the strain, the unloaded resistance, and the gauge factor. The calculated nonlinearity error as a percentage of the span could then be reduced in part due to the increase in the span, caused by an increased *GF* or *R*_0_, rather than solely due to a decrease in the absolute error value. To investigate this hypothesis, a correlation analysis is performed between the span and the absolute nonlinearity error. Figure 9 provides a plot of the correlation, including a linear regression line.

The analysis reveals a strong linear relationship between the absolute nonlinearity error and the sensor span, as evidenced by the Pearson correlation coefficient of 0.989. The linear regression provides the following expression for this relationship, with an R^2^ value of 97.9%:

Absolute nonlinearity error=4.325+0.2152×Span

The slope of this effect of 0.2152 confirms that an increase in sensitivity, which leads to an increased span, would increase the absolute nonlinearity error to a lesser degree than the span. This then effectively leads to a reduced relative nonlinearity error, since this relative error is calculated by dividing the absolute error by the span.

This is an interesting finding, as it suggests that the absolute nonlinearity error is largely a function of the span, and might at first glance not appear to be directly sensitive to the selected process parameters. However, because the selection of parameters affects the span, e.g., via their influences on the gauge factor, opportunities exist to indirectly reduce the relative nonlinearity error. This can be achieved by selecting parameters that increase the span by increasing the sensitivity, i.e., the gauge factor or piezoresistive coefficient. This can in fact be accomplished by changing the process parameters, e.g., by increasing the infill angle [20,25]. It can thus be said that the nonlinearity error can be affected by the selected process parameters, although the effect may be indirect.

### 3.2. Hysteresis

**Figure 10:**
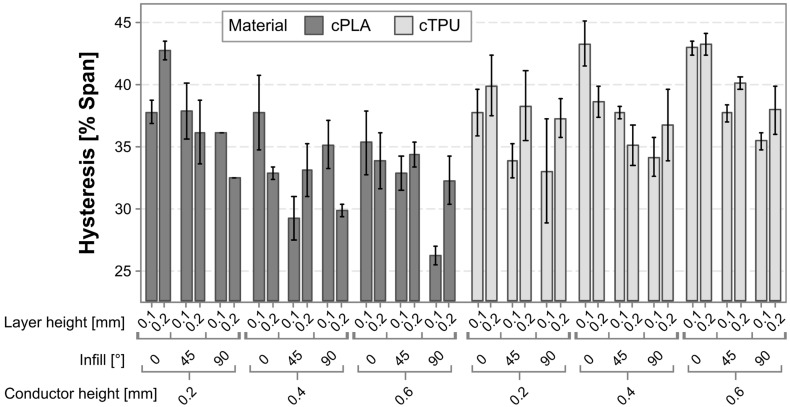
Bar chart of the hysteresis exhibited by each of the tested sensor variants.

Figure 10 shows a graphical representation of the hysteresis error for all tested sensors. A first observation is that the error contribution from hysteresis is larger than that from nonlinearity, which is in agreement with findings in existing literature on additively manufactured sensors [29]. Secondly, the materials respond differently to changes in the conductor height, with cPLA showing a reduced hysteresis for a greater conductor height, while cTPU does not. Both materials show a decreasing hysteresis error as the angle of the infill pattern is increased.

**Figure 11:**
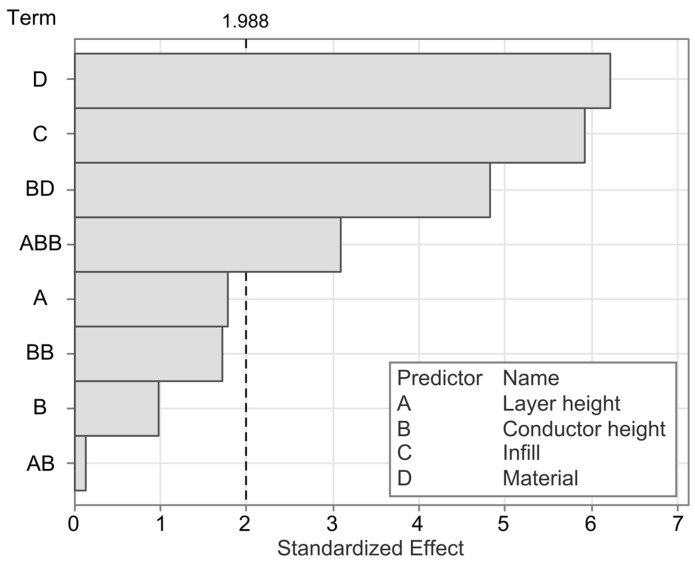
Pareto chart of the significant effects on the sensor hysteresis. The dashed line indicates the a = 0.05 significance level.

To better understand the factors and interaction effects contributing to the hysteresis, a linear regression model is employed. The model has an R^2^ value of 58.78%, indicating that most, but not all, influencing factors are captured in the experimental variables. The Pareto chart in Figure 11 shows that the most significant factors affecting the hysteresis are the material, the infill angle, and the interaction between material and conductor height. There is a further statistically significant interaction effect between the layer height and the conductor height squared.

**Figure 12:**
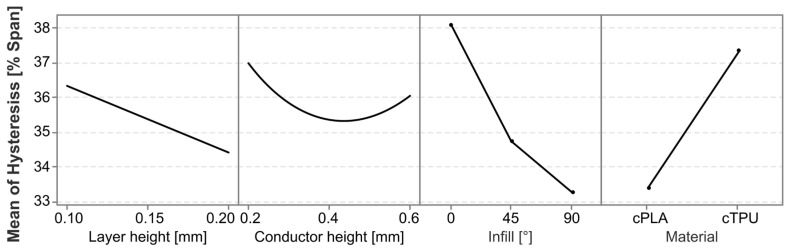
Main effects plot for the factors impacting the hysteresis error.

**Figure 13:**
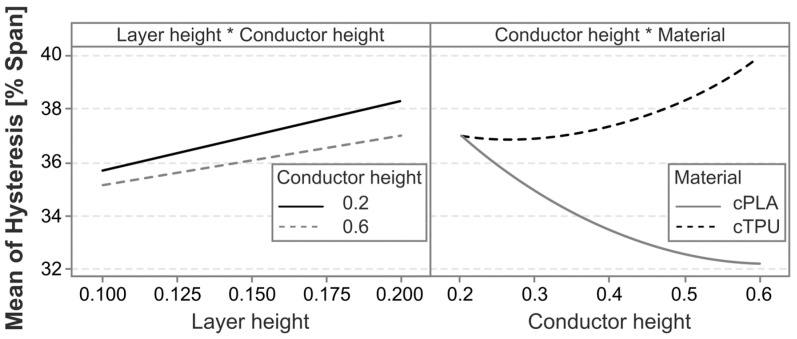
Interaction plots for the statistically significant interaction effects on the hysteresis error. The * symbol indicates an interaction between the two factors.

To further analyze the different statistically significant effects and their directionality, the main effects and interaction effects are plotted in Figure 12 and Figure 13, respectively. The sensor hysteresis is most significantly affected by the material, followed by the infill pattern. The cPLA sensors on average exhibited a significantly lower hysteresis than those produced from cTPU. The interaction between the material and the conductor height shows a marked difference between the two CPCs, with cPLA exhibiting a reduced hysteresis as the conductor height increases, whereas cTPU shows an increased hysteresis under the same conditions.

**Figure 14:**
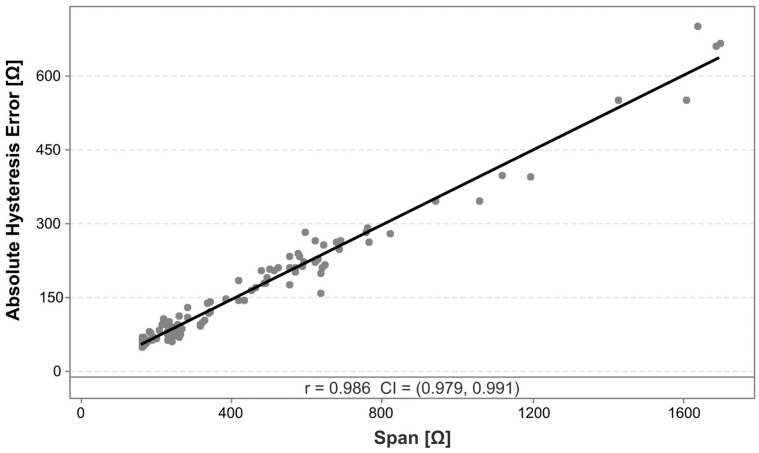
Correlation analysis between the absolute hysteresis error and the span, including a linear regression approximation. The indicated values for r and CI represent the Pearson correlation coefficient and the 95% confidence interval, respectively.

Klimm et al. [53] show how hysteresis in CPC-based sensors can be related to time-dependent transverse deformations in viscoelastic materials, particularly due to non-constant Poisson’s ratios. The differences in hysteresis between the two CPCs could then potentially be caused by different deformation behavior of the matrices, or by matrix–filler interactions [68]. For both materials, sensors produced with a higher infill angle exhibit a significantly reduced hysteresis. As this is a similar effect as that observed for the nonlinearity error, a similar correlation analysis is employed to test for a potential correlation between the span and the absolute hysteresis error, as shown in Figure 14.

The analysis reveals a strong correlation between the absolute hysteresis error and the span, with a Pearson correlation coefficient of 0.986. A linear regression finds that the relationship can be described by the following formula:

Absolute hysteresis error=−6.278+0.3799×Span

with an R^2^ of 97.2%. It is therefore likely that the relative hysteresis error, similar to the nonlinearity error, is indirectly sensitive to changes in sensor design and process parameters. Specifically, parameters that achieve a high sensitivity, such as using a 90° infill pattern, are associated with a reduction in the relative hysteresis error, due to a span that increases more rapidly than the absolute error values do.

Lastly, the plot of the interaction effect between the layer height and the conductor height in Figure 13 shows effects that are nearly parallel. This means that, although the interaction effect is statistically significant, it is likely not the most suitable candidate to adjust when attempting to produce sensors with minimal hysteresis.

### 3.3. Drift

**Figure 15:**
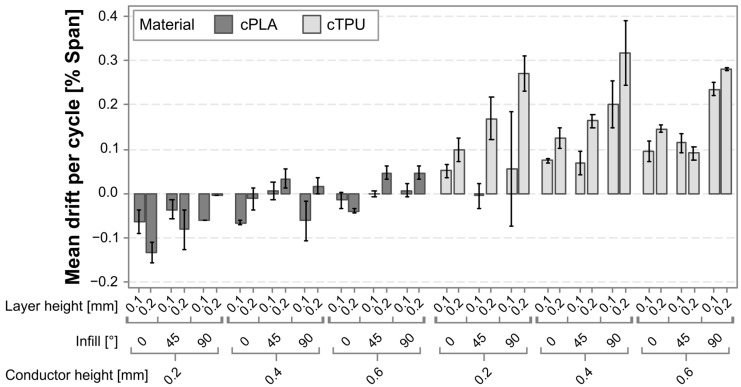
Bar chart of the mean drift per cycle for each of the tested sensor variants, based on the first ten load cycles.

Figure 15 provides a graphical representation of the relative drift per cycle for each of the investigated sensor types. The error values in this case are considerably lower than those for nonlinearity and hysteresis. However, it should be considered that although the different error types are on the same scale, this error value should not be directly compared due to the drift having a cumulative nature, with error contributions accumulating over multiple cycles. Secondly, it is worth noting that certain parameter combinations result in a negative drift value. As a negative drift is a sensor behavior that is equally as undesirable as a positive drift, the goal when selecting suitable parameters should not be the lowest value, but rather to obtain a drift slope of zero, which certain combinations do appear to achieve.

The drift values for cPLA are on average lower than those for cTPU. Both materials show an increasing drift as the conductor height is increased. There also appears to be a trend in the layer height, with the sensors produced with a 0.2 mm layer height typically exhibiting higher drift values. Lastly, the drift values seem to increase with an increased infill angle in both materials, although the effect appears to be more pronounced for cTPU.

**Figure 16:**
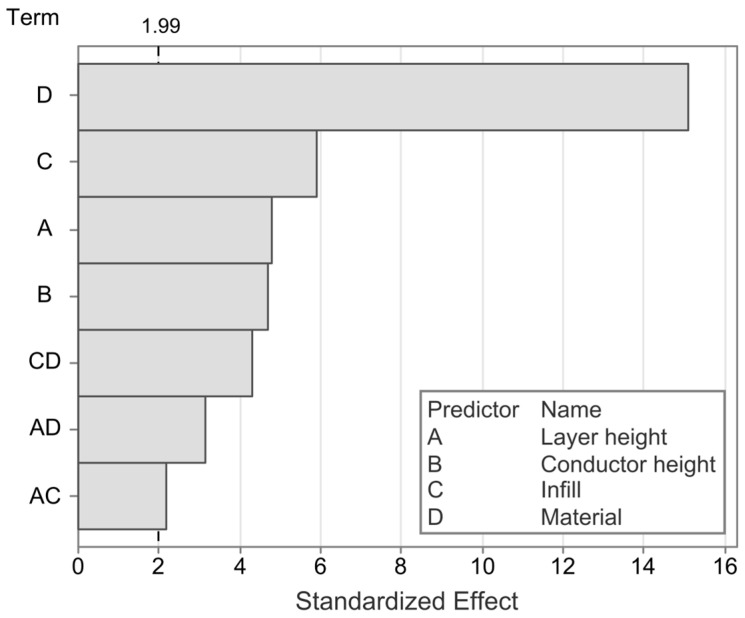
Pareto chart of the factors impacting the drift in the tested sensors. The dashed vertical line at 1.99 indicates the a = 0.05 significance level.

A linear regression model is applied to gain deeper insight into the statistically significant factors and interaction effects. The model has an R^2^ value of 80.16%, indicating that the majority of effects are captured by the model. The Pareto chart, as shown in Figure 16, confirms the previously observed significant impacts of material, infill, layer height, and conductor height. Furthermore, it reveals statistically significant interaction effects between the material and both the infill and the layer height, as well as an interaction between the layer height and the infill.

**Figure 17:**
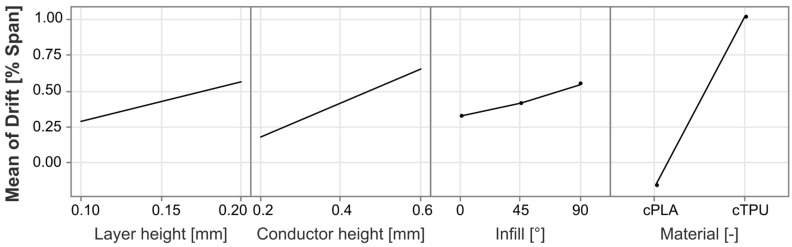
Main effects plot for the factors impacting the drift.

**Figure 18:**
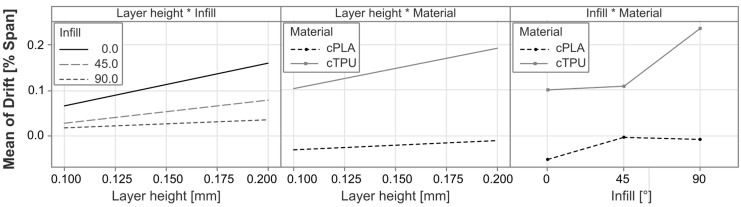
Interaction plots for the statistically significant interaction effects on the drift. The * symbol indicates an interaction between the two factors.

To further analyze the different statistically significant effects and their directionality, the main effects and interaction effects are plotted in Figure 17 and Figure 18, respectively.

The most significant impact on the drift is found in the selected material, with cPLA being capable of achieving near zero drift with certain process parameter combinations, whereas the drift for cTPU is typically in the positive range. The second most significant impact factor is the infill angle, with parts with a larger infill angle exhibiting a higher drift than those with a 0° infill, and cTPU showing particularly higher drift at a 90° infill, which is not present for cPLA. As the drift increases with the infill angle, a further analysis is once more carried out but reveals no strong correlation between the span and the absolute drift error values. A possible explanation for the observed behavior could be that the parts with higher infill angles have more strand-to-strand interfaces that are affected under load. The percolation network at these locations may be less dense, which could lead not only to a higher sensitivity [20,25] but also to an inhibited restoration of the percolation network as the load is removed. Such time-bound de-percolation and re-percolation effects under strain have previously been shown to exist in ethylene propylene diene monomer (EPDM) CPCs with carbon black filler [69], and may be responsible for similar drift behavior in the materials investigated in this work. The interaction effect between material and infill angle also aligns with this hypothesis, as existing works have found the sensor resistance and sensitivity to be more strongly affected by the infill angle in cTPU than in cPLA [20]. A further significant influence is exerted by the conductor height, where an increase in the factor is associated with an increase in the drift. This result is in agreement with findings in existing literature [29]. A positive relationship was also found for the layer height, for which a comparable study does not exist, to the authors’ knowledge. The interaction effect between layer height and material reveals that in cPLA, the influence of the layer height is very limited, whereas cTPU responds more strongly. This effect may be related to the resistance of cTPU being more strongly affected by the layer height than that of cPLA [20].

Although both materials respond similarly to changes in the design and process parameters, the fact that cPLA tends to exhibit negative drift, while cTPU tends to have a positive drift, means they require different design and processing approaches to achieve zero drift. The most suitable parameter combinations for cTPU use a 0° infill pattern, in combination with a low conductor and layer height. Even with these combinations, a limited positive drift is to be expected. For cPLA, a similar combination of settings would lead to an undesirable negative drift behavior. Therefore, this material would exhibit superior drift performance with a 0.4 mm or 0.6 mm conductor height, for which the combination with a 0.1 mm layer height and 45° infill achieve drift values closest to the desired zero slope.

## 4. Conclusions

The current study investigated the piezoresistive properties of sensors manufactured using material extrusion additive manufacturing. Specifically, it analyzed how the material, layer height, conductor height, and infill angle affect the nonlinearity error, hysteresis, and drift.

The relative nonlinearity error was shown to be influenced by the interaction effect between material selection and conductor height, with cPLA exhibiting lower error values than cTPU, except for the sensor variants with the lowest height. The observed significant increase in the nonlinearity error as the infill angle increases prompted a further analysis. This analysis revealed a strong correlation between the absolute nonlinearity error and the span of the sensor. In combination with the results in existing literature [20,25], it is hypothesized that the nonlinearity error may, in part, not be affected directly by the selected process parameters. Rather, the selected parameters affect sensor properties such as the gauge factor and unloaded resistance. These in turn affect the span of the sensor, and thereby indirectly influence the relative nonlinearity error. The values found for the nonlinearity error ranged from 18.51 to 26.69% for cPLA and from 20.91 to 27.98% for cTPU. This means an appropriate selection of parameters could affect up to a 30.7% and 25.3% reduction in nonlinearity error in cPLA and cTPU, respectively. The lowest nonlinearity error could be achieved by using the cPLA material and selecting a combination of parameters that lead to a high gauge factor, e.g., by using a 90° infill.

The hysteresis error was shown to be larger than the nonlinearity error. The magnitude of the hysteresis error was firstly dependent on the material selection, with cTPU exhibiting more hysteresis than cPLA. Secondly, the hysteresis depended significantly on the chosen infill angle, with increasing angles being related to a reduction in the error. Further analysis suggests that the hysteresis exhibited, in part, a similar indirect sensitivity to process parameters as found for the nonlinearity error. This means that parameters that lead to an increase in the span, such as by increasing the gauge factor, are associated with a lower relative hysteresis error. The hysteresis error was found to range from 26.22 to 42.80% in cPLA and from 33.01 to 43.34% for cTPU. This means the parameter selection could provide up to a 38.7% and 23.8% reduction in hysteresis in cPLA and cTPU, respectively. The lowest hysteresis error was achieved using the cPLA material and a 90° infill angle.

The error related to drift was most significantly affected by the selected material, with cPLA exhibiting lower drift than cTPU. Some of the investigated cPLA-based sensors exhibited a negative drift, which is a behavior that is equally as undesirable as a positive drift. Unlike the nonlinearity and hysteresis, absolute drift errors did not correlate with the span. While both cPLA and cTPU exhibited a similar response to the design and process parameters, they required different parameters to approach a zero-drift behavior due to their tendency to need a positive or negative adjustment, respectively. The best suited combination for cTPU was a 0° infill pattern, with a lower conductor height and layer height. The cPLA material performed best with a 45° infill combined with a 0.1 mm layer height and either a 0.4 mm or a 0.6 mm conductor height. However, considering the positive effects of using a 90° infill on sensor sensitivity [20,25], nonlinearity and hysteresis, and the limited difference in drift for cPLA sensors between using 45° and 90° infill angles, the possibility of selecting a 90° infill should also be considered a valid option for this material.

Of the two investigated materials, the cPLA material typically exhibits more favorable sensing properties compared to the cTPU material, i.e., lower nonlinearity and hysteresis errors, and the ability to reach near-zero drift with certain combinations of process parameters. Despite this, the use of cTPU or similar materials in sensor applications may still be relevant, as the material offers other potential benefits, such as the ability to withstand far larger deformations than the cPLA material is capable of. This property could enable sensing in, e.g., soft robotics applications, where cPLA would be unsuited due to its mechanical properties.

In all investigated sensors, it should be noted that the nonlinearity and hysteresis errors were quite significant, typically exceeding 20% of the span. This finding was not unexpected, as such behavior is common in CPC sensors [29]. Applications requiring a high measurement accuracy may therefore require additional measures to adequately address the various types of measurement error, beyond what can be achieved through process parameters alone. In such cases, other solutions should be considered, for example, by minimizing measurement errors through the use of multi-sensor setups undergoing opposite load cases [35,70]. For measurements with less stringent accuracy requirements, e.g., detecting whether a robotic gripper has successfully contacted an object [71], the directly achievable accuracy levels may be sufficient. In such cases, using the findings in this work to minimize undesirable properties can allow for direct improvements to sensor characteristics.

The current work presented an analysis of three of the most important error types in additively manufactured piezoresistive sensors, but opportunities for future works to further characterize the behavior of such sensors remain. Firstly, the R^2^ values for the nonlinearity and hysteresis models, of 61.97% and 58.78%, respectively, suggest that some influencing factors were not yet considered during the test planning. Further experimental research could be of value in identifying and understanding these influencing factors. Secondly, future works could focus on further sensor aspects, such as the frequency response, response time, and sensitivity to environmental conditions, including humidity, temperature or electromagnetic interference. Such investigations could provide a deeper understanding of additively manufactured sensors, thereby supporting their development and implementation in practical applications.

## Figures and Tables

**Table 1:** Process parameters for each of the materials used in the experiment [20].

Setting	Unit	TPU	cPLA	cTPU
Extrusion temperature	°C	235	235	235
Speed	mm/s	20	15	20
Extrusion multiplier	-	1.02	0.94	1.05
Trace width	mm	0.4	0.4	0.4
Shells	-	2	0	0
Bed temperature	°C	40	40	40

## Data Availability

The dataset will be made available on request from the authors.

## Abbreviations

The following abbreviations are used in this manuscript:

AM	Additive manufacturing
BFSL	Best fit straight line
CI	Confidence interval
CPC	Conductive polymer composite
cPLA	Conductive polylactic acid
cTPU	Conductive thermoplastic polyurethane
EPDM	Ethylene propylene diene monomer
GF	Gauge factor
PLA	Polylactic acid
TPU	Thermoplastic polyurethane
r	Pearson correlation coefficient
R_0_	Initial unloaded sensor resistance
SEM	Scanning electron microscope
